# Trajectory Tracking Using Cumulative Risk–Sensitive Finite Impulse Response Filters

**DOI:** 10.3390/mi16040365

**Published:** 2025-03-22

**Authors:** Yi Liu, Shunyi Zhao

**Affiliations:** Key Laboratory of Advanced Process Control for Light Industry, Ministry of Education, Jiangnan University, Wuxi 214000, China; shunyi@jiangnan.edu.cn

**Keywords:** trajectory tracking, risk sensitive, FIR filters, robust filters

## Abstract

Trajectory tracking is a critical component of autonomous driving and robotic motion control. This paper proposes a novel robust finite impulse response (FIR) filter for linear time-invariant systems, aimed at enhancing the accuracy and robustness of trajectory tracking. To address the limitations of infinite impulse response (IIR) filters in complex environments, we integrate a cumulative risk–sensitive criterion with an FIR structure. The proposed filter effectively mitigates model mismatches and temporary modeling uncertainties, making it highly suitable for trajectory tracking in dynamic and uncertain environments. To validate its performance, a comprehensive vehicle trajectory tracking experiment is conducted. The experimental results demonstrate that, compared to the Kalman filter (KF), risk–sensitive filter (RSF), and unbiased FIR (UFIR) filter, the proposed algorithm significantly reduces the average tracking error and exhibits superior robustness in complex scenarios. This work provides a new and effective solution for trajectory tracking applications, with broad potential for practical implementation.

## 1. Introduction

With the continuous advancements in automatic control, sensor technology, and communication systems, trajectory tracking has become increasingly prevalent in intelligent systems, including autonomous driving and robotic motion. Trajectory data are generally collected by global navigation satellite system (GNSS) devices. During the acquisition process, issues such as information latency and missing data may occur [1]. These problems arise due to various factors, including high maneuverability of the vehicle, network failures, and measurement errors [2,3]. State estimation serves as a fundamental approach to addressing the challenges inherent in trajectory tracking [4].

State estimation refers to a method of estimating the state of a system based on its measured outputs. Filtering is a form of state estimation. In this context, filtering does not refer to frequency-domain filtering but rather to time-domain filtering based on state-space equations. It aims to estimate the system’s internal state using noisy measurements while minimizing estimation errors, ensuring accuracy and robustness in dynamic systems. In previous research, numerous state estimation methods have been proposed. Among these, the most renowned is the Kalman filter (KF) [5]. The KF addresses the optimal estimation problem for linear time-invariant systems with known parameters, where both system noise and observation noise are Gaussian. Due to its simplicity and computational efficiency, KF has been widely applied. By employing a straightforward target model, KF can be designed to optimally track maneuvering vehicles, including aircraft, ships, and submarines, within tactical systems [6]. When the system is nonlinear, the performance of the KF significantly deteriorates. To address nonlinear problems, the extended KF (EKF) for nonlinear systems and the unscented KF (UKF) for highly nonlinear systems are derived [7,8]. An EKF using forward-looking infrared sensor data enhances target tracking by leveraging size, shape, motion, and atmospheric jitter, excelling in close-range air-to-air missile tracking despite clutter [9]. Research in [10] uses the EKF to estimate the state of a moving target detected by a single unmanned aerial vehicle and predicts the optimal trajectory using the EKF model, demonstrating that prediction accuracy significantly improves when the model accurately reflects the target dynamics. Some studies adopt hybrid methods combining the KF with other techniques to achieve better and faster tracking results. Research in [11] uses a hybrid method of camshift method as the main tracking technique and KF for prediction and correction.

However, the KF and its extended variants belong to the category of infinite impulse response (IIR) estimation, which inherently suffers from several limitations. These include limited robustness against model mismatches, increased sensitivity to temporary modeling uncertainties, and strong dependence on initial conditions and noise statistics [5,12,13,14]. These drawbacks highlight the need for alternative estimators, such as finite impulse response (FIR) estimators, which rely on a finite set of recent measurements for state estimation. FIR estimators offer improved robustness against temporary modeling uncertainties and ensure BIBO stability, albeit at the cost of higher computational complexity—a challenge mitigated by advancements in computational technology. The robustness of the KF and unbiased FIR (UFIR) filter has been evaluated under various conditions in [15], demonstrating that the UFIR filter outperforms the KF in scenarios involving inaccuracies in noise statistics, model mismatches, and temporary uncertainties. Research in [16] presents the extended least square UFIR filter, a nonlinear state estimator that provides consistent performance under uncertain noise modeling, outperforming the traditional EKF and particle filter, and it is suitable for tracking applications using the constant velocity model. In [17], experimental results further confirm that the UFIR filter for vehicle tracking over wireless sensor networks with delayed and missing data surpasses the performance of the KF and $H_{\infty }$ filter. In [18], the research proposes a variational Bayesian-based scheme for monitoring sensor noise covariance in nonlinear state space, achieving better estimation accuracy, with its effectiveness verified through localization and rotary flexible joint experiments. In [19], FIR filters outperform the KF in object tracking under high noise conditions. An iterative FIR filter for nonlinear systems is proposed, which improves target tracking by using unscented transformation and variational Bayesian for better accuracy and robustness with a reduced computational load [20]. The research in [21] presents a novel state estimation method that combines the UFIR filter and KF using transfer learning to handle parameter uncertainties and noise statistics, improving robustness and performance in target tracking.

In summary, robust filters have demonstrated significant potential in addressing trajectory tracking challenges. In [22], an FIR filter integrating the instantaneous risk–sensitive criterion with the FIR estimator is introduced. This filter, termed the instantaneous risk–sensitive FIR (IRSFIR) filter, exhibits superior robustness compared to conventional FIR filters. Building upon the risk–sensitive yet distinct criterion from the IRSFIR filter, we introduce a cumulative risk–sensitive cost function that accounts for the total estimation error accumulated from the initial time to the present within the estimation horizon. The main contributions of this paper are summarized as follows:

1. This paper introduces a new robust FIR filter based on a cumulative risk–sensitive cost function, which accounts for the total estimation error accumulated from the initial time to the present moment within the estimation horizon by reformulating the complex exponential cost function into a tractable max–min optimization problem.
2. To validate the proposed approach, a trajectory tracking experiment is conducted to assess the performance of four filters. The experimental results demonstrate that the proposed cumulative risk–sensitive FIR (CRSFIR) filter outperforms the KF, risk–sensitive filter (RSF), and UFIR filter in terms of trajectory tracking accuracy.

The remainder of this paper is structured as follows: Section 2 derives the extended state-space model and formulates the problem. Section 3 provides a detailed derivation of the CRSFIR filter. Section 4 presents the experimental results and analysis. Finally, Section 5 concludes this paper.

## 2. Extend State–Space Model and Problem Formulation

Consider a linear discrete time–invariant system, which is represented as

(1) $$\begin{array}{cc} x_{k} & =Fx_{k-1}+Eu_{k}+Bw_{k}, \end{array}$$

(2) $$\begin{array}{cc} y_{k} & =Hx_{k}+v_{k}, \end{array}$$

where *k* is the discrete time index, $x_{k}\in R^{K}$ is the state vector, $u_{k}\in R^{L}$ is the input vector, $y_{k}\in R^{P}$ is the measurement vector, and $F\in R^{K\times K}$, $E\in R^{K\times L}$, $B\in R^{K\times M}$, and $H\in R^{P\times K}$ are the state transition matrix, input matrix, noise matrix, and observation matrix, respectively. The system noise $w_{k}\in R^{M}$ and the measurement noise $v_{k}\in R^{P}$ are zero–mean white Gaussian and mutually uncorrelated. The covariances of $w_{k}$ and $v_{k}$ are denoted by *Q* and *R*, respectively.

To derive the CRSFIR filter, the state–space equations (Equations (1) and (2)) are extended over a horizon [m,k] consisting of *N* points, where m=k−N+1. The extended models are represented as

(3) $$\begin{array}{cc} X_{m,k} & =F_{N}x_{m}+S_{N}U_{m,k}+D_{N}W_{m,k}, \end{array}$$

(4) $$\begin{array}{cc} Y_{m,k} & =H_{N}x_{m}+L_{N}U_{m,k}+G_{N}W_{m,k}+V_{m,k}, \end{array}$$

where the extended vectors are specified as

(5) $$\begin{array}{cc} X_{m,k} & ={\left[x_{m}^{⊤}\cdots x_{k}^{⊤}\right]}^{⊤}, \end{array}$$

(6) $$\begin{array}{cc} Y_{m,k} & ={\left[y_{m}^{⊤}\cdots y_{k}^{⊤}\right]}^{⊤}, \end{array}$$

(7) $$\begin{array}{cc} U_{m,k} & ={\left[u_{m}^{⊤}\cdots u_{k}^{⊤}\right]}^{⊤}, \end{array}$$

(8) $$\begin{array}{cc} W_{m,k} & ={\left[w_{m}^{⊤}\cdots w_{k}^{⊤}\right]}^{⊤}, \end{array}$$

(9) $$\begin{array}{cc} V_{m,k} & ={\left[v_{m}^{⊤}\cdots v_{k}^{⊤}\right]}^{⊤}, \end{array}$$

where the symbol ⊤ represents the transpose operation. The extended matrices are represented by

(10) $$\begin{array}{cc} F_{N} & ={\left[I^{⊤}\;F^{⊤}\;\cdots \;{\left(F^{N-2}\right)}^{⊤}\;{\left(F^{N-1}\right)}^{⊤}\right]}^{⊤}, \end{array}$$

(11) $$\begin{array}{cc} S_{N} & =\left[\begin{array}{ccccc} E & 0 & \cdots & 0 & 0\\ FE & E & \cdots & 0 & 0\\ \vdots & \vdots & \ddots & \vdots & \vdots \\ F^{N-1}E & F^{N-2}E & \cdots & FE & E \end{array}\right], \end{array}$$

(12) $$\begin{array}{cc} H_{N} & ={\bar{H}}_{N}F_{N}, \end{array}$$

(13) $$\begin{array}{cc} L_{N} & ={\bar{H}}_{N}S_{N}, \end{array}$$

(14) $$\begin{array}{cc} G_{N} & ={\bar{H}}_{N}D_{N}, \end{array}$$

in which ${\bar{H}}_{N}=\mathrm{diag}\left(H\cdots H\right)$. The symbol $D_{N}$ can be represented as $S_{N}$ by replacing *E* with *B*, and the symbol *I* is the identity matrix. The noise term $G_{N}W_{m,k}+V_{m,k}$ in (4) can be shown to be zero–mean with the covariance $\Lambda_{N}$, which is represented by

(15) $$\begin{array}{c} \Lambda_{N}=G_{N}Q_{N}G_{N}^{⊤}+R_{N}, \end{array}$$

where $Q_{N}=\mathrm{diag}\left(Q\cdots Q\right)$ and $R_{N}=\mathrm{diag}\left(R\cdots R\right)$. The current state $x_{k}$ can be represented in terms of the state $x_{m}$, the input vectors $U_{m,k}$, and the noise vectors $W_{m,k}$ as

(16) $$\begin{array}{c} x_{k}=F^{N-1}x_{m}+{\bar{S}}_{N}U_{m,k}+{\bar{D}}_{N}W_{m,k}, \end{array}$$

where ${\bar{S}}_{N}$ and ${\bar{D}}_{N}$ are the last row vectors of $S_{N}$ and $D_{N}$, respectively.

In [22], the instantaneous risk–sensitive criterion is defined as

(17) $$\begin{array}{c} J^{\prime }=-\frac{2}{\theta }\log E\left[\exp\left\{-\frac{\theta }{2}{\left(x_{k}-{\hat{x}}_{k}^{\prime }\right)}^{⊤}\left(x_{k}-{\hat{x}}_{k}^{\prime }\right)\right\}\right], \end{array}$$

where θ<0 represents the risk–sensitive parameter, the symbol E denotes the expectation operator, and ${\hat{x}}_{k}^{\prime }$ corresponds to the state estimate of the IRSFIR filter, derived from measurements over the horizon [m,k]. Building upon (17), we introduce a cumulative formulation of the risk–sensitive criterion. In contrast to the instantaneous risk–sensitive criterion, the cumulative risk–sensitive criterion is defined as follows:

$$J=-\frac{2}{\theta }\log E\left[\exp\left\{-\frac{\theta }{2}\Psi_{s,k}\right\}\right],$$

(18) $$\Psi_{s,k}=\sum_{l=s}^{k-1}{\left(x_{l}-{\hat{x}}_{l}^{\prime }\right)}^{⊤}\left(\cdots \right)+{\left(x_{k}-{\hat{x}}_{k}\right)}^{⊤}\left(x_{k}-{\hat{x}}_{k}\right),$$

where m<s<k. Here, the index *s* denotes the starting point of error accumulation, the horizon length of [s,k] is $N_{1}$, ${\hat{x}}_{k}$ represents the state estimate of the CRSFIR filter at time *k*, and $x_{l}^{\prime }$ corresponds to the state estimate of the IRSFIR filter at time *l*, computed using measurements collected over the interval [m,l]. The notation (⋯) indicates the same term as its preceding counterpart.

The primary distinction between the instantaneous and cumulative criteria lies in the structure of the exponential term. Specifically, the instantaneous criterion incorporates only the error at the current time step within its exponential term. In contrast, the cumulative criterion considers the aggregation of errors over the interval [s,k]. This cumulative approach depends on prior estimates derived from the interval [s,k−1], which are computed using the IRSFIR filter. As demonstrated in (18), measurements within [s,k] are directly employed, while those within [m,s] are indirectly integrated through IRSFIR filter estimates. Consequently, the final estimate ${\hat{x}}_{k}$ effectively utilizes all measurements across the range [m,k].

The problem can be formulated as follows: Given the models in (1) and (2), develop a new FIR filter by minimizing the criterion defined in (18).

## 3. Cumulative Risk–Sensitive FIR Filter

In (18), the constant term $-\frac{2}{\theta }$ does not influence the extremum–seeking process and can thus be omitted. Given that the exponential function is monotonically increasing, its omission does not alter the extremum–seeking behavior. Consequently, the cost function (18) can be simplified as

(19) $$\begin{array}{c} J=E\left[\exp\left\{-\frac{\theta }{2}\Psi_{s,k}\right\}\right]. \end{array}$$

Equation (19) is mathematically equivalent to (18) when optimizing for the extremum. In (18), the accumulation of errors initiates at time *s*. Consequently, in the extended state–space equations, the subscript *m* should be substituted with *s*, and the interval length adjusted from *N* to $N_{1}$. Here, *E* denotes the expectation operator, and (19) can be reformulated as an integral operation. The expectation of the exponential quadratic cost function (19) can be evaluated using the joint probability density functions of $x_{s}$, $W_{s,k}$, and $V_{s,k}$. Accordingly, the joint probability density function $p(x_{s},W_{s,k},V_{s,k})$ is represented as

(20) $$\begin{array}{c} p\left(x_{s},W_{s,k},V_{s,k}\right)=\eta \exp\left\{-\frac{1}{2}j_{k}\right\}, \end{array}$$

where η represents the constant term and $j_{k}$ is given by

(21) $$\begin{array}{c} j_{k}={\left(x_{s}-\bar{m}\right)}^{⊤}{\bar{P}}^{-1}\left(x_{s}-\bar{m}\right)+W_{s,k}^{⊤}Q_{N_{1}}^{-1}W_{s,k}+V_{s,k}^{⊤}R_{N_{1}}^{-1}V_{s,k}, \end{array}$$

where $\bar{m}$ and $\bar{P}$ are the mean and covariance of $x_{s}$, and they can be computed by the least mean square criterion [23]. Specifically, $\bar{m}$ and $\bar{P}$ can be represented as

(22) $$\begin{array}{cc} \bar{m} & ={\left(H_{N_{1}}^{⊤}\Lambda_{N_{1}}^{-1}H_{N_{1}}\right)}^{-1}H_{N_{1}}^{⊤}\Lambda_{N_{1}}^{-1}\left(X_{s,k}-L_{N_{1}}U_{s,k}\right), \end{array}$$

(23) $$\begin{array}{cc} \bar{P} & ={\left(H_{N_{1}}^{⊤}\Lambda_{N_{1}}^{-1}H_{N_{1}}\right)}^{-1}. \end{array}$$

The simplified cost function can be computed as

(24) $$\begin{array}{cc} \; & E\left[\exp\left\{-\frac{\theta }{2}\Psi_{s,k}\right\}\right]\\ \; & =\eta \int \exp\left\{-\frac{\theta }{2}\Psi_{s,k}\right\}p\left(x_{s},W_{s,k},V_{s,k}\right)\;dx_{s}\;dW_{s,k}\;dV_{s,k}\\ \; & =\eta \int \exp\left\{-\frac{\theta }{2}\Psi_{s,k}\right\}\exp\left\{-\frac{1}{2}j_{k}\right\}\;dx_{s}\;dW_{s,k}\;dV_{s,k}\\ \; & =\eta \int \exp\left\{-\frac{1}{2}{\bar{j}}_{k}\right\}\;dx_{s}\;dW_{s,k}\;dV_{s,k}, \end{array}$$

where ${\bar{j}}_{k}=\theta \Psi_{s,k}+j_{k}$. To simplify (24), a useful mathematical result can be applied [24]. Specifically, the integral

$$\int \exp\left\{-\frac{1}{2}{\bar{j}}_{k}\right\}\;dx_{s}\;dW_{s,k}\;dV_{s,k}$$

is finite if and only if ${\bar{j}}_{k}$ attains a minimum over the domain $\left\{x_{s},W_{s,k},V_{s,k}\right\}$. Under this condition, the value of the integral is proportional to

$$\begin{array}{c} \exp\left\{-\frac{1}{2}\min_{x_{s},W_{s,k},V_{s,k}}{\bar{j}}_{k}\right\}. \end{array}$$

Meanwhile, based on (4), $V_{s,k}$ can be expressed in terms of $x_{s}$ and $W_{s,k}$. Consequently, the criterion (24) can be further reduced to

(25) $$\begin{array}{c} \eta \int \exp\left\{-\frac{1}{2}{\bar{j}}_{k}\right\}\;dx_{s}\;dW_{s,k}\;dV_{s,k}\;\;\propto \;\;\eta \;\exp\left\{-\frac{1}{2}\min_{x_{s},W_{s,k}}{\bar{j}}_{k}\right\}. \end{array}$$

Based on (24) and (25), the minimization of the criterion (19) can ultimately be simplified to

(26) $$\begin{array}{c} \max_{{\hat{x}}_{k}}\min_{x_{s},W_{s,k}}{\bar{j}}_{k}, \end{array}$$

where ${\bar{j}}_{k}$ can be represented as

(27) $$\begin{array}{cc} {\bar{j}}_{k} & =j_{k}+\theta \Psi_{s,k}\\ \; & ={\left(x_{s}-\bar{m}\right)}^{⊤}{\bar{P}}^{-1}\left(x_{s}-\bar{m}\right)+W_{s,k}^{⊤}Q_{N_{1}}^{-1}W_{s,k}\\ \; & +{\left(Y_{s,k}-H_{N_{1}}x_{s}-L_{N_{1}}U_{s,k}-G_{N_{1}}W_{s,k}\right)}^{⊤}R_{N_{1}}^{-1}\left(\cdots \right)\\ \; & +\theta \left({\left(x_{s}-{\hat{x}}_{s}^{\prime }\right)}^{⊤}\left(\cdots \right)+\cdots +{\left(x_{k}-{\hat{x}}_{k}\right)}^{⊤}\left(x_{k}-{\hat{x}}_{k}\right)\right). \end{array}$$

By substituting (22) and (23) into (27), ${\bar{j}}_{k}$ can be represented in a compact form as

(28) $$\begin{array}{c} {\bar{j}}_{k}=A_{k}^{⊤}B_{k}A_{k}, \end{array}$$

where $A_{k}$ and $B_{k}$ are represented, respectively, by

(29) $$\begin{array}{c} A_{k}={\left[\begin{array}{ccccccc} x_{s}^{⊤} & W_{s,k}^{⊤} & {\hat{x}}_{k}^{⊤} & \cdots & {\hat{x}}_{s}^{\prime ⊤} & Y_{s,k}^{⊤} & U_{s,k}^{⊤} \end{array}\right]}^{⊤}, \end{array}$$

(30) $$\begin{array}{c} B_{k}=\left[\begin{array}{ccccccc} B_{k}^{1,1} & B_{k}^{1,2} & B_{k}^{1,3} & B_{k}^{1,4} & \cdots & B_{k}^{1,N_{1}+3} & B_{k}^{1,N_{1}+4}\\ ⋆ & B_{k}^{2,2} & B_{k}^{2,3} & B_{k}^{2,4} & \cdots & B_{k}^{2,N_{1}+3} & B_{k}^{2,N_{1}+4}\\ ⋆ & ⋆ & B_{k}^{3,3} & B_{k}^{3,4} & \cdots & B_{k}^{3,N_{1}+3} & B_{k}^{3,N_{1}+4}\\ ⋆ & ⋆ & ⋆ & B_{k}^{4,4} & \cdots & B_{k}^{4,N_{1}+3} & B_{k}^{4,N_{1}+4}\\ \vdots & \vdots & \vdots & \vdots & \ddots & \vdots & \vdots \\ ⋆ & ⋆ & ⋆ & ⋆ & \cdots & B_{k}^{N_{1}+3,N_{1}+3} & B_{k}^{N_{1}+3,N_{1}+4}\\ ⋆ & ⋆ & ⋆ & ⋆ & \cdots & ⋆ & B_{k}^{N_{1}+4,N_{1}+4} \end{array}\right]. \end{array}$$

Specific expressions for each block of the matrix $B_{k}$ can be found in Appendix A.

According to (29), we define *a*, *b*, and *c* as

(31) $$\begin{array}{cc} a & ={\left[\begin{array}{cc} x_{s}^{⊤} & W_{s,k}^{⊤} \end{array}\right]}^{⊤},\;b={\hat{x}}_{k},\\ c & ={\left[\begin{array}{ccccc} {\hat{x}}_{k-1}^{\prime ⊤} & \cdots & {\hat{x}}_{s}^{\prime ⊤} & Y_{s,k}^{⊤} & U_{s,k}^{⊤} \end{array}\right]}^{⊤}. \end{array}$$

Then, the blocks of $B_{k}$ can be decomposed into the following submatrices:

(32) $$\begin{array}{cc} C^{2,2} & =B_{k}^{3,3},\\ C^{1,1} & =\left[\begin{array}{cc} B_{k}^{1,1} & B_{k}^{1,2}\\ ⋆ & B_{k}^{2,2} \end{array}\right],\;C^{1,2}=\left[\begin{array}{c} B_{k}^{1,3}\\ B_{k}^{2,3} \end{array}\right],\\ C^{1,3} & =\left[\begin{array}{cccc} B_{k}^{1,4} & \cdots & B_{k}^{1,N_{1}+3} & B_{k}^{1,N_{1}+4}\\ B_{k}^{2,4} & \cdots & B_{k}^{2,N_{1}+3} & B_{k}^{2,N_{1}+4} \end{array}\right],\\ C^{2,3} & =\left[\begin{array}{cccc} B_{k}^{3,4} & \cdots & B_{k}^{3,N_{1}+3} & B_{k}^{3,N_{1}+4} \end{array}\right],\\ C^{3,3} & =\left[\begin{array}{cccc} B_{k}^{4,4} & \cdots & B_{k}^{4,N_{1}+3} & B_{k}^{4,N_{1}+4}\\ \vdots & \ddots & \vdots & \vdots \\ ⋆ & \cdots & B_{k}^{N_{1}+3,N_{1}+3} & B_{k}^{N_{1}+3,N_{1}+4}\\ ⋆ & \cdots & ⋆ & B_{k}^{N_{1}+4,N_{1}+4} \end{array}\right], \end{array}$$

where the symbol ⋆ represents the symmetric counterpart of the corresponding block within the matrix structure. According to (31) and (32), Equation (28) can be rewritten in a simpler form as

(33) $$\begin{array}{c} {\bar{j}}_{k}={\left[\begin{array}{c} a\\ b\\ c \end{array}\right]}^{⊤}\left[\begin{array}{ccc} C^{1,1} & C^{1,2} & C^{1,3}\\ C^{2,1} & C^{2,2} & C^{2,3}\\ C^{3,1} & C^{3,2} & C^{3,3} \end{array}\right]\left[\begin{array}{c} a\\ b\\ c \end{array}\right], \end{array}$$

where $C^{2,1}$, $C^{3,1}$, and $C^{3,2}$ are the transpose matrices of $C^{1,2}$, $C^{1,3}$, and $C^{2,3}$, respectively. To solve the optimization problem (26), the following useful result can be applied [24]. When the inequality conditions

$$C^{1,1}>0,\;C^{2,2}-{\left(C^{1,2}\right)}^{⊤}{\left(C^{1,1}\right)}^{-1}C^{1,2}<0$$

are satisfied, the optimal values of *a* and *b*, which minimize ${\bar{j}}_{k}$ with respect to *a* and maximize ${\bar{j}}_{k}$ with respect to *b*, exist and are represented by

(34) $$\begin{array}{c} \left[\begin{array}{c} a\\ b \end{array}\right]=-{\left[\begin{array}{cc} C^{1,1} & C^{1,2}\\ C^{2,1} & C^{2,2} \end{array}\right]}^{-1}\left[\begin{array}{c} C^{1,3}\\ C^{2,3} \end{array}\right]c. \end{array}$$

Then, the solution of (26) is given as

(35) $$\begin{array}{c} {\hat{x}}_{k}=-\gamma {\left[\begin{array}{cc} C^{1,1} & C^{1,2}\\ {\left(C^{1,2}\right)}^{⊤} & C^{2,2} \end{array}\right]}^{-1}\left[\begin{array}{c} C^{1,3}\\ C^{2,3} \end{array}\right]\left[\begin{array}{c} {\hat{x}}_{k-1}^{\prime }\\ \vdots \\ {\hat{x}}_{s}^{\prime }\\ Y_{s,k}\\ U_{s,k} \end{array}\right], \end{array}$$

where γ=[0⋯0I] is a coefficient matrix designed to extract the ${\hat{x}}_{k}$ component from Equation (34). Equation (35) indicates that the computation of ${\hat{x}}_{k}$ requires the prior calculation of ${\hat{x}}_{s}^{\prime },\ldots ,{\hat{x}}_{k-1}^{\prime }$. The complete CRSFIR filter algorithm is outlined in Algorithm 1.
**Algorithm 1** CRSFIR filter algorithm.
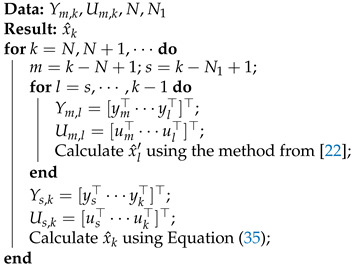


As outlined in Algorithm 1, the CRSFIR filter operates through two primary phases. Initially, the estimates produced by the IRSFIR filter prior to time *k* are aggregated. Subsequently, these estimates are incorporated into the solving formula (35) to derive the CRSFIR filter estimate at time *k*. Although the inclusion of IRSFIR filter computations elevates the overall computational complexity of the algorithm, the CRSFIR filter substantially enhances estimation performance by considering accumulated estimation errors over a defined time interval.

## 4. GNSS–Based Tracking of a Moving Vehicle

**Figure 1:**
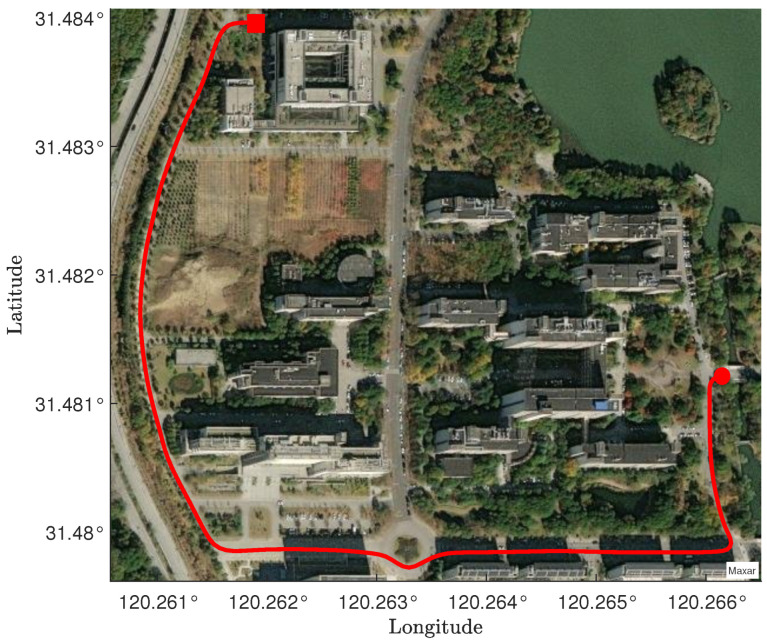
The first path segment: vehicle trajectory under satellite imagery. The red square marks the start of the experiment, and the red circle indicates the end of the experiment.

**Figure 2:**
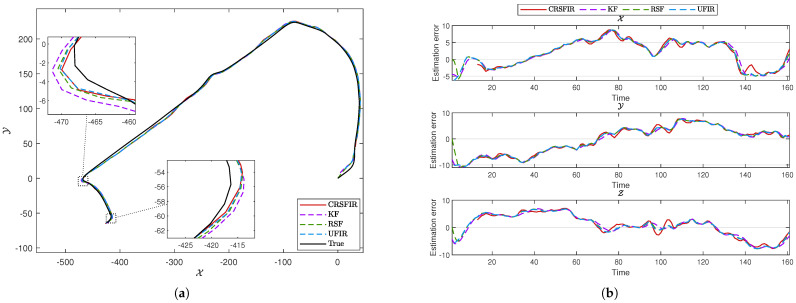
(**a**) Vehicle trajectories generated by the cumulative risk–sensitive FIR (CRSFIR) filter, Kalman filter (KF), risk–sensitive filter (RSF), and unbiased FIR (UFIR) filter. (**b**) Estimation error curves generated by the CRSFIR filter, KF, RSF, and UFIR filter along the X, Y, and Z axes.

To evaluate the performance of the CRSFIR filter in trajectory tracking, we conducted an experiment focused on tracking the trajectory of a moving vehicle. The experiment comprises two distinct trajectory segments. The first segment features fewer turns and primarily consists of straight–line motion, with minimal surrounding buildings. In contrast, the second segment involves more frequent turns and a higher density of surrounding buildings, creating a more complex environment. A GNSS processing unit is responsible for parsing the transmitted satellite signals to obtain precise location data. The sampling interval τ during the experiment is set to 1 s. The raw data collected by the GNSS are initially obtained from the World Geodetic System 1984 (WGS84) coordinate system. To facilitate the evaluation of the filtering algorithm’s performance, WGS84 coordinates are converted to the Earth–centered, Earth–fixed (ECEF) coordinate system. The ECEF is a Cartesian system with three axes, X, Y, and Z, measured in meters. The experiment uses a simplified trajectory tracking model, with the coefficient matrices being represented as

$$\begin{array}{c} F=\left[\begin{array}{cccccc} 1 & \tau & 0 & 0 & 0 & 0\\ 0 & 1 & 0 & 0 & 0 & 0\\ 0 & 0 & 1 & \tau & 0 & 0\\ 0 & 0 & 0 & 1 & 0 & 0\\ 0 & 0 & 0 & 0 & 1 & \tau \\ 0 & 0 & 0 & 0 & 0 & 1 \end{array}\right],\;B=\left[\begin{array}{ccc} \frac{1}{2}\tau^{2} & 0 & 0\\ \tau & 0 & 0\\ 0 & \frac{1}{2}\tau^{2} & 0\\ 0 & \tau & 0\\ 0 & 0 & \frac{1}{2}\tau^{2}\\ 0 & 0 & \tau \end{array}\right],\;H=\left[\begin{array}{cccccc} 1 & 0 & 0 & 0 & 0 & 0\\ 0 & 0 & 1 & 0 & 0 & 0\\ 0 & 0 & 0 & 0 & 1 & 0 \end{array}\right], \end{array}$$

and E=0. The state matrix is represented as ${\left[\begin{array}{cccccc} p_{X}, & v_{X}, & p_{Y}, & v_{Y}, & p_{Z}, & v_{Z} \end{array}\right]}^{⊤}$. The contents of the state matrix represent the positions and velocities in the X, Y, and Z directions, respectively. The system noise covariance is given by $Q=0.2I_{3\times 3}$, and the measurement noise covariance is given by $R=0.8I_{3\times 3}$. To validate the performance of the CRSFIR filter, which is designed for linear systems, it is compared with other linear filtering methods. The KF and UFIR filter are selected as representative examples of IIR and FIR approaches, respectively. Additionally, the RSF, known for its robustness, is included in the comparison to further evaluate the effectiveness of the CRSFIR filter under varying conditions. The risk–sensitive parameter θ=−1. The horizon length N=13 and $N_{1}=3$. The choice of the horizon length is not fixed and can also take other values. However, since the horizon length directly affects whether the filter constraints can be satisfied, extensive simulation and testing are required to determine a suitable value. Figure 1 shows the true trajectory of the first path segment in the experiment. The experiment starts at the location marked by the red square and ends at the location marked by the red circle. In Figure 2a, the vehicle trajectories generated by the four filters are shown. It is clearly observed that the tracking performance of all four filters is satisfactory. From a global perspective, there is no significant superiority or inferiority among the four filters. By zooming in on two turning points in the figure, it becomes clear that the CRSFIR filter demonstrates superior tracking performance. In Figure 2b, the figure shows the estimation error curves generated by the CRSFIR filter, KF, RSF, and UFIR filter along the X, Y, and Z axes. Table 1 lists the root mean square error (RMSE) of the four filters in the process. For the first path segment, the RMSE along the X-axis shows minimal differences among the four filters. The CRSFIR filter outperforms both the KF and UFIR filter, ranking second only to the RSF. In the Y and Z axes, the CRSFIR filter exhibits markedly better performance than the other three filters. Overall, the CRSFIR filter exhibits the best performance in terms of trajectory tracking accuracy. The computation times for the four filters are 3.3620 s (CRSFIR), 0.0034 s (KF), 0.0110 s (RSF), and 0.0125 s (UFIR), respectively. As expected, the computation time of the CRSFIR filter is the longest.

**Figure 3:**
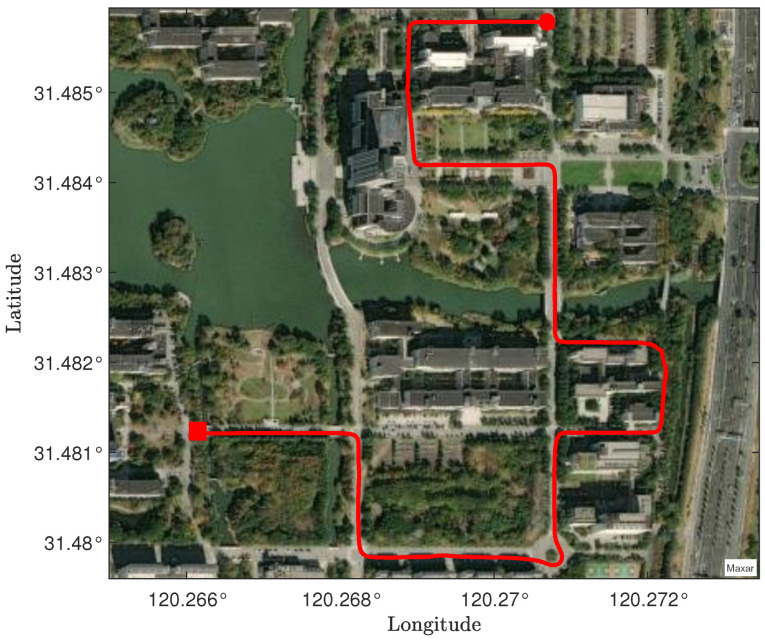
Second path segment: vehicle trajectory under satellite imagery. The red square marks the start of the experiment, and the red circle indicates the end of the experiment.

**Figure 4:**
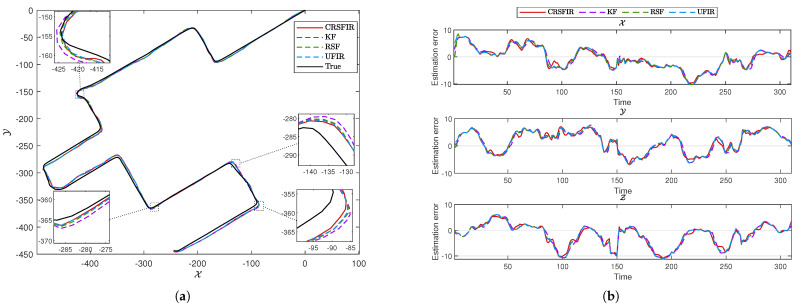
(**a**) Vehicle trajectories generated by the CRSFIR filter, KF, RSF, and UFIR filter. (**b**) Estimation error curves generated by the CRSFIR filter, KF, RSF, and UFIR filter along the X, Y, and Z axes.

Figure 3 shows the true trajectory of the second path segment in the experiment. As depicted in Figure 3, the second path segment features more turns and is surrounded by more buildings compared to the first segment, making it more challenging for trajectory tracking. Such an environment is typical in real–world scenarios, making it well suited for evaluating the robustness of the CRSFIR filter in trajectory tracking. In Figure 4a, the vehicle trajectories generated by the four filters are displayed, with magnified views at four turning points. It is evident that the CRSFIR filter estimate aligns more closely with the true trajectory compared to the other filters. Figure 4b presents the estimation errors of the four filters. The error curves of the four filters exhibit minor differences, but the CRSFIR filter clearly converges faster in certain regions, highlighting the advantage of FIR filters over IIR filters. Table 2 lists the RMSE values of the four filters in the second path segment. As shown in Table 2, the RMSE of the CRSFIR filter is significantly lower than that of the KF, RSF, and UFIR filter in all three directions. This indicates that the CRSFIR filter exhibits superior robustness in trajectory tracking under complex environments. The computation times of the four filters in the second path segment are 9.1711 s (CRSFIR), 0.0047 s (KF), 0.0174 s (RSF), and 0.0193 s (UFIR), respectively. This result is similar to that in the first path segment, since CRSFIR initially calculates ${\hat{x}}_{s}^{\prime }\cdots {\hat{x}}_{k-1}^{\prime }$, which consumes a significant amount of time.

Based on the two trajectory tracking examples mentioned above, it can be concluded that the CRSFIR filter outperforms the KF, RSF, and UFIR filter in terms of tracking accuracy, while also demonstrating superior robustness.

## 5. Conclusions

This paper proposes a novel robust FIR filter based on a cumulative risk–sensitive criterion, distinguishing it from conventional filters that rely on instantaneous criteria. The cumulative criterion considers all estimation errors from the initial time to the current time within the estimation horizon, providing a more comprehensive evaluation of performance. By integrating the risk–sensitive criterion into the FIR estimation framework, the proposed filter achieves enhanced robustness compared to traditional IIR and FIR filters. To validate its performance in trajectory tracking, a vehicle trajectory tracking experiment is conducted under realistic environmental conditions. Experimental results demonstrate that the CRSFIR filter outperforms the KF, RSF, and UFIR filter in terms of tracking accuracy. However, its significantly longer computation time limits its applicability to offline estimation rather than real–time applications. There are several aspects of the filter algorithm that can be further improved, including enhancing computational efficiency, developing adaptive algorithms, conducting a more comprehensive convergence analysis, and extending its application to nonlinear systems.

## Figures and Tables

**Table 1:** Root mean square error (RMSE) of different algorithms.

	CRSFIR	KF	RSF	UFIR
X	4.2313	4.2400	4.2114	4.2471
Y	4.7536	5.3023	5.1775	5.2463
Z	4.1208	4.1778	4.1367	4.1564

**Table 2:** RMSE of different algorithms.

	CRSFIR	KF	RSF	UFIR
X	3.7002	3.9892	3.9818	3.9933
Y	4.0199	4.2116	4.1798	4.1647
Z	4.5875	4.6798	4.6747	4.6727

## Data Availability

https://github.com/lyi61/Trajectory-Tracking-Using-Cumulative-Risk-Sensitive-FIR-Filters_code_and_data.git.

## Appendix A

Specific expressions for each block of the matrix $B_{k}$ are expressed as follows:

$$\begin{array}{cc} B_{k}^{1,1} & ={\bar{P}}^{-1}+H_{N_{1}}^{⊤}R_{N_{1}}^{-1}H_{N_{1}}+\theta \sum_{i=0}^{N_{1}-1}{\left(F^{i}\right)}^{⊤}F^{i}, \end{array}$$

$$\begin{array}{cc} B_{k}^{1,2} & =H_{N_{1}}^{⊤}R_{N_{1}}^{-1}G_{N_{1}}+\theta \sum_{i=0}^{N_{1}-1}{\left(F^{i}\right)}^{⊤}{\bar{B}}_{i}{\bar{I}}_{i+1}, \end{array}$$

$$\begin{array}{cc} B_{k}^{1,i} & =-\theta {\left(F^{N_{1}-i+2}\right)}^{⊤},\;\mathrm{where}\;3\leq i\leq N_{1}+2, \end{array}$$

$$\begin{array}{cc} B_{k}^{1,N_{1}+3} & =-{\bar{P}}^{-1}T_{N_{1}}-H_{N_{1}}^{⊤}R_{N_{1}}^{-1}, \end{array}$$

$$\begin{array}{cc} B_{k}^{1,N_{1}+4} & =-{\bar{P}}^{-1}T_{N_{1}}L_{N_{1}}+H_{N_{1}}^{⊤}R_{N_{1}}^{-1}L_{N_{1}}+\theta \sum_{i=0}^{N_{1}-1}{\left(F^{i}\right)}^{⊤}{\bar{E}}_{i}{\bar{I}}_{i+1}, \end{array}$$

$$\begin{array}{cc} B_{k}^{2,2} & =Q_{N_{1}}^{-1}+G_{N_{1}}^{⊤}R_{N_{1}}^{-1}G_{N_{1}}+\theta \sum_{i=0}^{N_{1}-1}{\bar{I}}_{i+1}^{⊤}{\bar{B}}_{i}^{⊤}{\bar{B}}_{i}{\bar{I}}_{i+1}, \end{array}$$

$$\begin{array}{cc} B_{k}^{2,i} & =-\theta {\bar{I}}_{N_{1}-i+3}^{⊤}{\bar{B}}_{N_{1}-i+2}^{⊤},\;\mathrm{where}\;3\leq i\leq N_{1}+2, \end{array}$$

$$\begin{array}{cc} B_{k}^{2,N_{1}+3} & =-G_{N_{1}}^{⊤}R_{N_{1}}^{-1}, \end{array}$$

$$\begin{array}{cc} B_{k}^{2,N_{1}+4} & =-G_{N_{1}}^{⊤}R_{N_{1}}^{-1}L_{N_{1}}+\theta \sum_{i=0}^{N_{1}-1}{\bar{I}}_{i+1}^{⊤}{\bar{B}}_{i}^{⊤}{\bar{E}}_{i}{\bar{I}}_{i+1}, \end{array}$$

$$\begin{array}{cc} B_{k}^{i,i} & =\theta I,\;\mathrm{where}\;3\leq i\leq N_{1}+2, \end{array}$$

$$\begin{array}{cc} B_{k}^{i,j} & =0,\;\mathrm{where}\;3\leq i<j\leq N_{1}+3, \end{array}$$

$$\begin{array}{cc} B_{k}^{i,N_{1}+4} & =-\theta {\bar{E}}_{N_{1}-i+2}{\bar{I}}_{N_{1}-i+3},\;\mathrm{where}\;3\leq i\leq N_{1}+2, \end{array}$$

$$\begin{array}{cc} B_{k}^{N_{1}+3,N_{1}+3} & =T^{⊤}{\bar{P}}^{-1}T+R_{N_{1}}^{-1}, \end{array}$$

$$\begin{array}{cc} B_{k}^{N_{1}+3,N_{1}+4} & =-T^{⊤}{\bar{P}}^{-1}TL_{N_{1}}-{\bar{P}}^{-1}L_{N_{1}}, \end{array}$$

$$\begin{array}{cc} B_{k}^{N_{1}+4,N_{1}+4} & =L_{N_{1}}^{⊤}T^{⊤}{\bar{P}}^{-1}TL_{N_{1}}+L_{N_{1}}^{⊤}R_{N_{1}}^{-1}L_{N_{1}}+\theta \sum_{i=0}^{N_{1}-1}{\bar{I}}_{i+1}^{⊤}{\bar{E}}_{i}^{⊤}{\bar{E}}_{i}{\bar{I}}_{i+1}. \end{array}$$

where $T={\left(H_{N_{1}}^{⊤}\Lambda_{N_{1}}^{-1}H_{N_{1}}\right)}^{-1}H_{N_{1}}^{⊤}\Lambda_{N_{1}}^{-1}$, and ${\bar{B}}_{i}$, ${\bar{E}}_{i}$, and ${\bar{I}}_{i}$ are defined, respectively, as

$$\begin{array}{cc} {\bar{B}}_{i} & =[F^{i}B\;F^{i-1}B\cdots B], \end{array}$$

$$\begin{array}{cc} {\bar{E}}_{i} & =[F^{i}E\;F^{i-1}E\cdots E], \end{array}$$

$$\begin{array}{cc} {\bar{I}}_{i} & =\underset{N_{1}}{\underset{︸}{\left[\mathrm{diag}\left(\overset{i}{\overset{︷}{I\;I\cdots I}}\right)\;0\cdots 0\right]}}. \end{array}$$
